# The crystal structure of human XPG, the xeroderma pigmentosum group G endonuclease, provides insight into nucleotide excision DNA repair

**DOI:** 10.1093/nar/gkaa688

**Published:** 2020-08-21

**Authors:** Rocío González-Corrochano, Federico M Ruiz, Nicholas M I Taylor, Sonia Huecas, Srdja Drakulic, Mercedes Spínola-Amilibia, Carlos Fernández-Tornero

**Affiliations:** Centro de Investigaciones Biológicas Margarita Salas, CSIC, Ramiro de Maeztu 9, 28040 Madrid, Spain; Centro de Investigaciones Biológicas Margarita Salas, CSIC, Ramiro de Maeztu 9, 28040 Madrid, Spain; Centro de Investigaciones Biológicas Margarita Salas, CSIC, Ramiro de Maeztu 9, 28040 Madrid, Spain; Centro de Investigaciones Biológicas Margarita Salas, CSIC, Ramiro de Maeztu 9, 28040 Madrid, Spain; Centro de Investigaciones Biológicas Margarita Salas, CSIC, Ramiro de Maeztu 9, 28040 Madrid, Spain; Centro de Investigaciones Biológicas Margarita Salas, CSIC, Ramiro de Maeztu 9, 28040 Madrid, Spain; Centro de Investigaciones Biológicas Margarita Salas, CSIC, Ramiro de Maeztu 9, 28040 Madrid, Spain

## Abstract

Nucleotide excision repair (NER) is an essential pathway to remove bulky lesions affecting one strand of DNA. Defects in components of this repair system are at the ground of genetic diseases such as xeroderma pigmentosum (XP) and Cockayne syndrome (CS). The XP complementation group G (XPG) endonuclease cleaves the damaged DNA strand on the 3′ side of the lesion coordinated with DNA re-synthesis. Here, we determined crystal structures of the XPG nuclease domain in the absence and presence of DNA. The overall fold exhibits similarities to other flap endonucleases but XPG harbors a dynamic helical arch that is uniquely oriented and defines a gateway. DNA binding through a helix-2-turn-helix motif, assisted by one flanking α-helix on each side, shows high plasticity, which is likely relevant for DNA scanning. A positively-charged canyon defined by the hydrophobic wedge and β-pin motifs provides an additional DNA-binding surface. Mutational analysis identifies helical arch residues that play critical roles in XPG function. A model for XPG participation in NER is proposed. Our structures and biochemical data represent a valuable tool to understand the atomic ground of XP and CS, and constitute a starting point for potential therapeutic applications.

## INTRODUCTION

The maintenance of genomic integrity is essential for cell function. Numerous agents including chemicals and radiation may cause DNA damage that, if unrepaired or repaired wrongly, generates mutations that can lead to disease. In order to avoid the formation of irreversible mutations, cells have developed various mechanisms that deal with the different types of DNA damage (1). The nucleotide excision repair (NER) system detects and removes DNA helix-distorting lesions produced by chemical or physical agents including UV-irradiation. When NER is defective, humans develop rare autosomal diseases such as xeroderma pigmentosum (XP), Cockayne syndrome (CS) and trichothiodystrophy, with symptoms ranging from high sun sensitivity and skin cancer risk to premature aging and neurological disorders (2,3). Depending on their role in XP disease, eight complementation groups (XPA-XPG and XPV) have been described, seven of which relate to proteins participating in NER (3).

Two NER sub-pathways have been defined, depending on initial recognition of the lesion (4,5). While in global-genome NER the XPC-RAD23 heterodimer identifies the lesion, transcribing RNA polymerases actively detect DNA damage in the so-called transcription-coupled NER. Both sub-pathways converge at the subsequent step, where the ten-subunit complex TFIIH is recruited to open and stabilize a repair bubble of about 24–32 nucleotides (6,7). TFIIH-mediated bubble opening is stimulated by both XPA and XPG, with the former binding to the junction between single and double-stranded DNA (ss/dsDNA junction) on the 5′side of the lesion (8,9). The undamaged strand is protected by the replication protein A (RPA) heterotrimer, while conformational changes reposition XPG and allow recruitment of the heterodimer formed by XPF and excision repair cross-complementing 1 (ERCC1), resulting in the formation of a pre-incision complex (10). The XPF nuclease cuts first on the 5′ side of the lesion, followed by 3′ cleavage by XPG (11). Coordinated with removal of the damaged fragment, the proliferating cell nuclear antigen (PCNA) fetches DNA polymerases to refill the gap (12).

XPG belongs to the family of flap endonucleases (FEN), which in mammals encompasses three additional members (13). FEN1 is involved in DNA replication, EXO1 participates in mismatch repair and double-strand break repair, while GEN1 resolves Holliday junctions. These enzymes comprise a catalytic domain that is compact in all cases except for XPG, which includes a ∼600-residue insertion at the so-called helical arch. This poorly-conserved insertion presents low complexity and has been shown to mediate contacts with TFIIH and RPA (14). The XPG nuclease domain is, thus, formed by the association of two subdomains that are distant in the amino acid sequence. The N-subdomain comprises residues 1–100 while the I-subdomain includes residues 760–990, approximately. In addition, flap endonucleases display C-terminal extensions of different lengths that mediate interactions with other proteins such as PCNA in the case of FEN1 and XPG (15).

Flap endonucleases recognize ss/dsDNA junctions formed at different stages of DNA processing (16). GEN1 recognizes and cleaves Holliday junctions, while the preferred FEN1 substrate is a nicked duplex with both 5′ and 3′ single-stranded flaps and the endonucleolytic substrate of EXO1 presents a short 5′ flap. The XPG substrate in NER is a DNA bubble with a 5′ overhang of about 25 nucleotides (17,18), derived from XPF-ERCC1 cleavage (11). Both artificial DNA bubbles lacking the 5′ overhang and splayed-arm DNAs mimicking half bubbles can be cleaved by XPG *in vitro* (19,20). Flap nucleases cut the strand with the 5′ overhang at the phosphodiester bond that links nucleotides +1 and +2 into dsDNA, which is known as the scissile bond (13).

While the crystal structure of human XPG is currently unknown, those of human FEN1, EXO1 and GEN1 in complex with different DNA substrates revealed how these enzymes recognize and cleave their specific DNA substrates (21–23). The three enzymes use a helix-2turn-helix (H2TH) motif (Pfam entry PF06831) to bind the non-cleaved strand around 6–8 nucleotides into the dsDNA region. This interaction, which is mediated by a K^+^ or Na^+^ ion, helps position the scissile bond into the active site, formed by seven conserved carboxylates. The first exposed base pair of dsDNA is bound by a hydrophobic wedge, located in the loop between two α-helices. Beyond the active site, the protruding single-stranded DNA (ssDNA) 5′ flap is clamped by two additional α-helices forming a helical arch. Functional studies have shown that in FEN1 the helical arch unfolds to thread the 5′ flap and then becomes fully ordered to clamp DNA and allow hydrolysis (24). In addition, FEN1 embraces the dsDNA beyond the 3′ flap using the hydrophobic wedge α-helices on one face of the DNA double helix, and a β-pin on the opposite face. Next to the hydrophobic wedge, an acid block that is unique in FEN1 binds the 3′ flap. The crystal structure of Rad2, the yeast homolog of XPG, in complex with DNA has been determined (25). While it exhibits a similar binding mode for dsDNA at the H2TH motif and around the active site, the role of other relevant structural domains remains undetermined. Moreover, the lack of structural information for human XPG obscures the molecular basis of essential genetic diseases such as XP and CS.

Here, we report four crystal structures of the human XPG nuclease domain (XPGn) in the absence and presence of DNA. The structures unveil the conformation of critical structural motifs and suggest how the enzyme may recognize DNA prior to nuclease cleavage. The helical arch adopts a different orientation as compared to other human flap nucleases, which is likely relevant for TFIIH interaction and DNA positioning before incision. We use mutational analysis to identify positively-charged residues in the helical arch that play a role in DNA binding and cleavage. Our results rationalize existing biochemical data and provide an atomic frame to understand the molecular basis of genetic diseases associated with NER.

## MATERIALS AND METHODS

### Protein expression

The cDNA of human XPG (UniProt entry P28715) was used to generate chimeric gene for XPGn, encoding for residues 1–112 fused to residues 750–990 through the Gly-Thr dipeptide. This chimeric gene was cloned into plasmids pET28 (Novagen), pET29 (Novagen) and pETM11 (EMBL) to produce three protein variants differing in the position and cleavability of the purification tag. In the first construct, pET28 was modified to substitute the thrombin cleavage site with the human rhinovirus 3C protease target sequence and the chimeric gene was cloned between the NdeI and XhoI sites, thus encoding for XPGn with a cleavable N-terminal His-tag (His-3C-XPGn) that, after protease cleavage, includes the Gly-Pro-His tripeptide before the N-terminal Met in XPGn. In the second construct, the chimeric gene was cloned between the NdeI and XhoI sites of pET29, encoding for XPGn with a non-cleavable C-terminal His-tag (XPGn-His). In the third construct, the chimeric gene was cloned after the Tobacco Etch Virus (TEV) protease so that, upon protease cleavage of the N-terminal His-tag (His-TEV-XPGn), the resulting protein lacks the N-terminal Met in the sequence of XPGn. Point and double mutants were prepared using standard methodologies.

The three constructs were transformed in *Escherichia coli* strain BL21 Star (DE3) pRare and expressed differently. His-3C-XPGn was expressed at 37°C in 2× YT medium after induction with 1 mM IPTG at an optical density of 0.6 at 600 nm and harvested after 6 h. For seleno-methionine labelling, cells were centrifuged prior to induction, resuspended in M9 medium supplemented with vitamin B1 and an amino acid cocktail as described (26) and, after 15 min, protein expression was induced with 1 mM IPTG. XPGn-His cells were grown at 37°C in autoinducible TB medium at 37°C until an optical density of 2.0 at 600 nm was reached, then harvested after 18 h incubation at 22°C. His-TEV-XPGn was expressed at 25°C in LB medium after induction with 1 mM IPTG at an optical density of 0.6 at 600 nm and harvested after 18 h.

### Protein purification

Harvested cells were resuspended in buffer A (50 mM Tris pH 7.5, 500 mM KCl, 20 mM imidazole, 2 mM β-mercaptoethanol) supplemented with 0.5% Tween-20 and EDTA-free protease inhibitors (Roche), and lysed by sonication. The lysate was clarified by centrifugation at 40 000g and 4°C for 30 min and the supernatant loaded on a HisTrap column (GE Healthcare) equilibrated in buffer A. The column was washed with 10 volumes of buffer A and the protein eluted with buffer B (50 mM Tris pH 7.5, 500 mM KCl, 500 mM imidazole, 2 mM β-mercaptoethanol) using a gradient over 12 column volumes. Fractions containing XPGn were pooled and dialyzed against buffer C (50 mM Tris pH 7.5, 50 mM KCl, 2 mM β-mercaptoethanol) overnight. For His-3C-XPGn the 3C protease was added prior to dialysis in a 1:100 mass ratio, while for His-TEV-XPGn the TEV protease was added after dialysis in a 1:3 mass ratio and incubated for 16 h. In both cases, the protease and non-cleaved XPGn were removed using a HisTrap column, following the described protocol with minor modifications. Subsequently, for the three fusion proteins, the sample was concentrated and applied to a Superdex 75 column (GE Healthcare) equilibrated in buffer D (20 mM Tris pH 7.5, 50 mM KCl, 2 mM DTT). Fractions containing XPGn were concentrated to about 30 mg/ml and stored at −80°C. Purified proteins of the three constructs are hereafter termed XPGn1, XPGn2 and XPGn3. The D812A mutants of XPGn1 and XPGn2 were used for crystallization, while wild-type and point mutants of XPGn3 were used for functional tests, as all attempts to crystallize the latter proved unsuccessful.

### Preparation of DNA substrates

Synthetic oligonucleotide (Metabion) sequences are shown in Supplementary Table S2. For crystallization and electrophoretic mobility shift assays, oligonucleotides were dissolved in H_2_O to a final concentration of 8 mM. Annealed DNAs were prepared by mixing 1:1 molar ratios of partially complementary strands in buffer E (20 mM Tris pH 7.5, 100 mM KCl), followed by incubation at 95°C for 10 min and slow cooling to 4°C. For nuclease activity tests, the Cy5-labeled and non-labeled oligonucleotides, i.e. DNA-Ya and Yb, were annealed in buffer F (20 mM Tris pH 7.5, 150 mM KCl) following the same protocol.

### Crystallization

Crystals of free XPGn1 were obtained by mixing equal volumes of the protein and reservoir buffer containing 25% polyethylene glycol (PEG) 3350, 100 mM citric acid pH 3.5 at 22°C. The crystals were cryoprotected by addition of glycerol to a final concentration of 25% and flash frozen in liquid N_2_. For the XPG-DNA structures, XPGn1 and DNA-1 were mixed in a 1:1.2 molar ratio at a final protein concentration of 20 mg/ml in buffer E, for 30 min at 22°C prior to crystallization. Crystals of this complex belonging to two different space groups were grown at 22°C in 5% PEG 3350, 50 mM sodium citrate pH 4.0 and cryoprotected in the same buffer containing 25% ethylene glycol before plunging into liquid N_2_. Crystals of free XPGn2 were obtained from an equimolar mix of the protein at 10 mg/ml in buffer G (20 mM Tris pH 7.5, 100 mM KCl, 20 mM MgCl_2_, 1 mM DTT) with splayed-arm DNA, incubated for 30 min at 22°C prior to crystallization in 25% PEG 3350, 100 mM Bis–Tris pH 6.5, 200 mM ammonium sulfate. The crystals, showing no density for DNA, were cryoprotected in the same buffer containing 25% ethylene glycol before plunging into liquid N_2_.

### Data collection and structure determination

Diffraction data were collected at 100K at beamlines PROXIMA-1, ID29 and XALOC at the SOLEIL, ESRF and ALBA synchrotrons, respectively. Crystallographic data were processed using XDS (27). A summary of the data collection and processing statistics is given in Table 1. The structure of the free XPGn was determined by SAD phasing using a highly-redundant dataset obtained from merging two selenomethionine derivative crystals. The resulting model was employed to solve the structures of native XPGn, either alone or in complex with DNA, using Phaser (28). Manual building was carried out in Coot (29) while Phenix (30) and Refmac (31) were used for refinement, with statistics included in Table 1. Stereochemical validation of the final model was performed with Molprobity (32) and structure figures were prepared using Pymol.

**Table 1.** Data collection and refinement statistics

	Free1 (F1)	Free2 (F2)	Complex1 (C1)	Complex2 (C2)
Wavelength (Å)	0.9786	0.9787	0.9795	0.9795
Resolution (Å)	67.6–2.9 (3.0–2.9)	47.8–2.5 (2.6–2.5)	45.8–3.5 (3.6–3.5)	49.7–3.1 (3.2–3.1)
Space group	*P*2_1_2_1_2_1_	*P*4_2_2_1_2	*I*422	*P*42_1_2
Unit cell (Å)	78.24, 89.56, 268.95	134.22, 134.22, 110.81	129.51, 129.51, 118.73	127.59, 127.59, 118.96
Total reflections	3 458 740 (349 796)	256 046 (28 832)	105 235 (25 414)	504 482 (91 701)
Unique reflections	42 886 (4403)	35 554 (3887)	6627 (1536)	18 419 (3269)
Multiplicity	80.6 (79.4)	7.2 (7.4)	15.9 (16.5)	27.4 (28.1)
Completeness (%)	100 (100)	99.8 (98.1)	99.8 (99.6)	99.9 (99.9)
Mean *I*/sigma(*I*)	13.1 (1.0)	14.4 (1.0)	11.1 (0.9)	11.9 (0.9)
R-pim	0.061 (1.072)	0.034 (0.826)	0.052 (0.933)	0.052 (1.203)
CC1/2	0.998 (0.528)	0.999 (0.439)	0.998 (0.827)	0.998 (0.587)
Wilson *B*-factor	73.01	69.08	132.70	110.52
Reflections used in refinement	42814 (4201)	35494 (3431)	6552 (618)	18305 (1749)
Reflections used for *R*-free	1071 (105)	888 (86)	331 (32)	551 (50)
*R*-work	0.239 (0.382)	0.203 (0.303)	0.265 (0.489)	0.247 (0.415)
*R*-free	0.280 (0.399)	0.237 (0.336)	0.291 (0.555)	0.302 (0.460)
CC(work)	0.928 (0.313)	0.957 (0.707)	0.962 (0.664)	0.956 (0.639)
CC(free)	0.831 (0.161)	0.939 (0.647)	0.922 (0.396)	0.862 (0.302)
Protomers in asymmetric unit	4	2	1	2
Non-hydrogen atoms	10 271	4823	2914	5860
Protein residues	1267	583	313	645
RMS(bonds)	0.014	0.002	0.004	0.003
RMS(angles)	1.88	0.55	0.70	0.56
Ramachandran favored (%)	96.19	97.00	97.05	95.88
Ramachandran allowed (%)	3.72	2.65	2.62	4.12
Ramachandran outliers (%)	0.08	0.35	0.33	0.00
Rotamer outliers (%)	3.79	1.96	0.00	0.00
Clashscore	9.79	3.57	7.74	4.01
Average *B*-factor	109.27	82.05	212.30	145.28
PDB code	6TUR	6TUS	6TUW	6TUX

Statistics for the highest-resolution shell are shown in parentheses.

### Nuclease activity tests

25 nM of Cy5-labelled DNA-Y was incubated with 2.5, 5, 25 and 50 nM wild-type or mutant XPGn in buffer H (25 mM Tris pH 7.5, 30 mM KCl, 2.5 mM β-mercaptoethanol) supplemented with 0.5 mM MnCl_2_ at 37°C for 60 min in a reaction volume of 20 μl. The reaction was stopped by addition of an equal volume of buffer J (90% [v/v] formamide, 50 mM EDTA, 0.1% [w/v] bromophenol blue) and heating for 10 min at 95°C. The samples (10 μl) were loaded onto 16% denaturing TBE–urea 7 M polyacrylamide gel, run for 1 h at 10 W and scanned with a Typhoon Trio (Amersham Biosciences). For quantification, gels were analyzed with the ImageJ-NIH software.

### Electrophoretic mobility shift assay

For electrophoretic mobility shift assays (EMSA), 2.5 μM of the DNA-2 substrate was incubated with 1, 2, 4 and 8 μM protein in buffer H supplemented with 15% glycerol at 25°C for 30 min in a reaction volume of 20 μl. The samples (20 μl) were loaded on a 7% native polyacrylamide gel, run at 100 V for 1 h and visualized with ethidium bromide in a GelDoc 2000 (Bio Rad). For quantification, gels were analyzed with the ImageJ-NIH software.

### Thermal shift assay

The SYPRO Orange dye (Invitrogen) was diluted with protein buffer D from an original 5000× stock solution. Experiments were performed in a iQ5 (BIO-RAD) real-time PCR, with a sample volume of 20 μl and a final protein concetration of 0.1 mg/ml. Samples were subjected to a temperature ramp of 3.0°C/min, from 20 to 90°C, and fluorescence was recorded at 570 nm.

## RESULTS

### Crystal structures of the XPG nuclease domain in the free state

**Figure 1.**
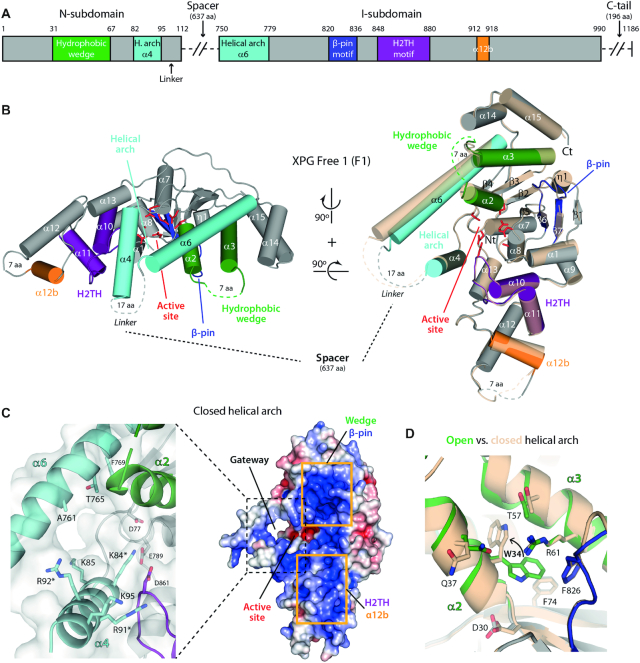
Crystal structure of XPGn in the DNA-free state. (**A**) Bar diagram of the structural motifs in the XPG nuclease domain. The spacer and the C-terminal tail, absent in our constructs, have been omitted for simplicity. (**B**) Crystal structures of F1 with open and closed conformations of the helical arch. Colors are as in panel A for the closed arch conformation, while the open arch conformation is shown in transparent cream color. (**C**) Surface charge representation (right) and close-up view of sidechains around the gateway (left) of F1 in the closed configuration of the helical arch. Residues mutated in this work are labeled with an asterisk. (**D**) Comparison of residue W34 position in the open (colors as in panel A) and closed (cream color) arch conformations of F1 molecules.

To investigate the structure of XPGn, we engineered a construct where the central spacer region (residues 113–749) and the C-terminal tail (991–1186) are absent (Figure 1A and Supplementary Figure S1). For crystallization, we introduced the D812A mutation to abolish the XPG nuclease activity (33). Two versions of the protein were purified that differ in the presence or absence of extra residues at the N-terminus (see Materials and Methods). Both constructs retain the ability to bind splayed-arm DNA (Supplementary Figure S2A) and were used to determine two crystal structures of XPGn in the free state, hereafter F1 and F2, with four and two molecules in the asymmetric unit, respectively (Table 1). XPGn is formed by a 7-stranded central β-sheet surrounded by 15 α-helices (Figure 1B). The structure presents an overall bean-like shape, reminiscent of other enzymes of the human flap endonuclease family (21–23), where structural elements of the family can be identified. The active site is located in a groove lying roughly at the center of the enzyme. The D812A mutation explains the absence of electron density for divalent cations in the active site, as this residue is required for metal coordination, together with six additional conserved carboxylates, i.e. D30, D77, E789, E791, D810 and D861 (Figure 1B, red). In further agreement, E791 in our structure presents an orientation that is incompatible with metal coordination (Supplementary Figure S3A, B).

Above the active site, a helical arch is formed by two α-helices that we labelled α4 (residues 82–97) and α6 (residues 750–779) in accordance with canonical nomenclature (22,23). In the full-length protein, α4 and α6 connect the N- and I-subdomains through the spacer region, while in our constructs they link through XPG residues 98–112 plus 2 extra amino acids derived from cloning, which all appear disordered (Figure 1A-B). This segment, thus, operates as a flexible linker allowing the helical arch to adopt its native configuration. Helices α4 and α6 are fully ordered in two F1 molecules, while the N-terminal half of α6 is disordered in the other two F1 molecules (Supplementary Figure S3C). In F2 crsytals, both the N-terminal half of α6 and the entire α4 are disordered (Supplementary Figure S3D). Comparison of F1 molecules where the arch is fully ordered shows that α6 can adopt two distinct conformations by pivoting on its C-terminal end (Figure 1B). In the open state, the α4 and α6 backbones lie ∼14 Å apart and define a gap of ∼11 Å, while in the closed state α6 approaches α4 by about 3 Å. This enables weak interactions at the outer rim of the arch, leading to the formation of a pore above the active site with a diameter of ∼9 Å (Figure 1C). The inner interface is contributed by basic residues K84, K85, R91 and R92 on the α4 side, and neutral residues on the α6 side. A similar pore in FEN1 was proposed to act as a gateway for 5′ flap threading prior to nuclease cleavage (24). Arch closure in the DNA-free state correlates with a striking conformational change in the hydrophobic core of the protein (Figure 1D). In the open state, residue W34 in α2 sits in a hydrophobic pocket that is sealed by a cation–π interaction with R61 in α3. In contrast, in the closed state, W34 flips into a looser hydrophobic environment and inserts between α3 and α6, which both shift in opposite directions.

The XPG nuclease domain contains additional structural motifs that are expected to interact with DNA according to surface charge distribution (Figure 1B-C). On one side of the active site, the H2TH motif (residues 848–880), comprising α10, α11 and their connecting loop, is flanked by helices α1 (residues 6–12) and α12b (residues 912–918). The latter is mainly composed by basic residues and connects with α12 through a linker that is partly disordered. On the other side of the active site, the hydrophobic wedge (residues 31–67) includes helix α2, which adopts different configurations due to W34 changes (see above), helix α3 and their connecting loop, which is mostly disordered. Finally, the β-pin motif (residues 820–836) comprises strands β6, β7 and the β6–β7 loop, which exhibits diverse conformations in the different DNA-free structures. The mobility of these structural motifs is likely to play a role in XPG function.

### Crystal structures of the XPG nuclease domain in complex with DNA

**Figure 2.**
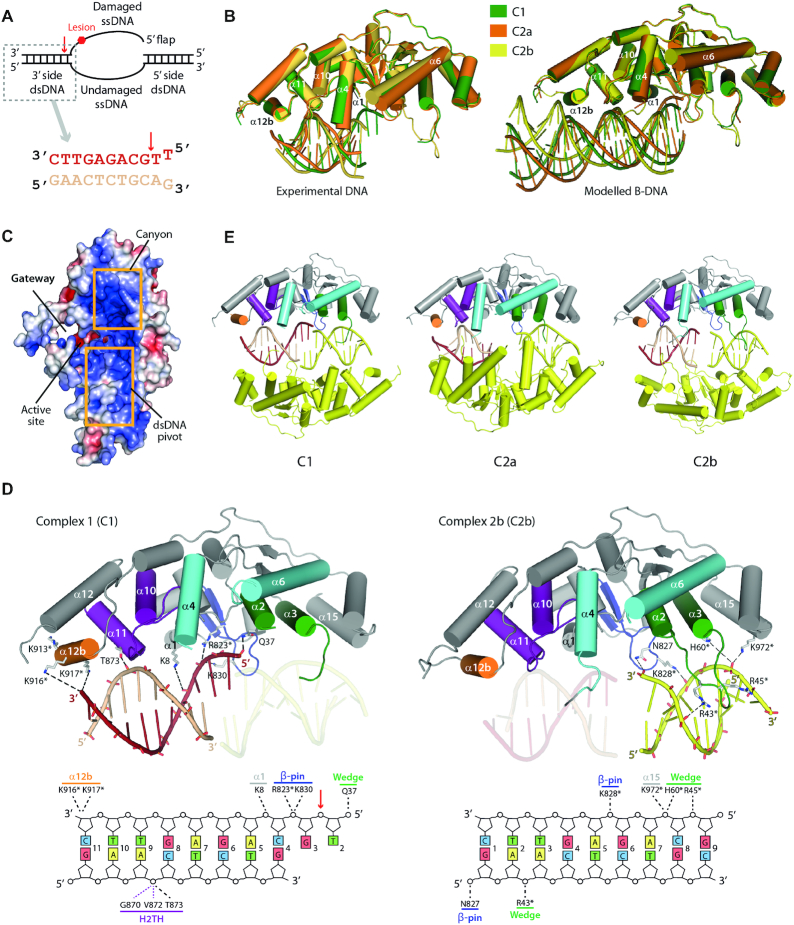
Crystal structure of XPGn in complex with DNA. (**A**) Schematic representation of the repair bubble and sequence of the crystallized DNA substrate. A red arrow indicates the scissile bond. (**B**) Crystal structures of the XPGn–DNA complexes superposed on the protein with experimental (left) and modelled (right) DNA. (**C**) Surface charge representation of C1 showing the main DNA interacting regions. (**D**) Above, crystal structures of C1 and C2b with depicted sidechains for residues in the vicinity of DNA. Below, schematic representation of DNA-interacting residues in XPG. Residues mutated in this work are marked with an asterisk. (**E**) Structures of XPGn in complex with DNA showing the symmetry-related XPGn–DNA complex in yellow.

To characterize the interaction between XPG and DNA, we obtained crystals of XPGn in complex with a short splayed-arm DNA mimicking the 3′ side of the repair bubble (Figure 2A). Two crystal forms were obtained with similar unit cell dimensions but different symmetry (Table 1). Crystals of the I422 space group contain one complex in the asymmetric unit, hereafter C1, while those belonging to space group *P*42_1_2 contain two distinct complexes, termed C2a and C2b. In all cases, the dsDNA region of the substrate exhibits clear density, while it is weaker or absent around the ss/dsDNA junction (Supplementary Figure S4). This may be due to the presence of the N-terminal methionine in the crystallized protein, which hampers access of the 5′ overhang into the active site. In agreement, nuclease activity tests show that this construct is unable to cleave splayed-arm DNA in conditions where XPGn lacking the N-terminal methionine is active (Supplementary Figure S2B). The protein structures derived from the three complexes exhibit the largest changes at the helical arch (Figure 2B). In all cases, the arch helices are fully ordered and adopt the closed state, though α6 in C2b is halfway tilted towards the open state. Interestingly, in the three complexes W34 lies within the hydrophobic pocket, suggesting that DNA binding is sufficient to close the helical arch and generate a gateway between α4 and α6 (Figure 2C).

Strikingly, while in the three complexes the DNA substrates contacts XPG around α11, the DNA trajectories significantly differ (Figure 2B). In both C1 and C2a, DNA binds XPG with the minor groove facing α11 and α12b, while the major groove faces α1. However, the DNA in C2a is tilted by 7° towards α1 and away from α12b, relative to C1. Furthermore, the DNA in C2b binds XPG in the opposite orientation, with the major groove facing α11 and α12b, while the minor groove faces α1. As compared to C1, its trajectory is tilted by 15° toward α12b and away from α1. Modeling of an ideal B-DNA on the experimental substrates shows clashes with the α12–α12b loop in the case of C2b or with the β-pin in the case of C2a (Figure 2B). In contrast, a straight B-DNA can be modeled on the C1 substrate with no clashes. Additionally, this peculiar orientation of the C1 substrate leads to partial DNA ordering in the vicinity of the active site, which allowed us to identify the damaged and undamaged DNA strands, as well as the putative scissile bond (Figure 2A).

As mentioned, the main contact involves the H2TH motif and the undamaged strand in the dsDNA region of the splayed-arm substrate (Figure 2D). The phosphate group located seven nucleotides into dsDNA from the putative scissile bond forms a hydrogen bond with the sidechain of T873 in α11. This contact is reinforced by the main chain of residues G870 and V872 in α11, which lie at hydrogen bond distance from the DNA backbone. Unexpectedly, the H2TH interaction is not mediated by a K^+^ ion in our structures, in spite of its presence in the crystallization buffer. Nuclease activity tests in the presence of different ions show that smaller monovalent ions such as Li^+^ allow splayed-arm cleavage in the same extent as K^+^ does (Supplementary Figure S2C). Besides, the dsDNA region that is most distal from the scissile bond contacts α12b, likely through basic residues K913, K916 and K917, all of which lie close to DNA. This additional interaction is predicted to confer XPG the possibility to bind longer dsDNA segments, in agreement with DNA protection experiments (34). Moreover, K8 in α1 lies next to the damaged strand within the dsDNA region in the vicinity of the active site, suggesting that this residue may participate in DNA positioning. Additionally, the damaged strand in C1 locates next to β-pin residues R823 and K830, which likely play a role in the interaction with the ss/dsDNA junction. The hydrophobic wedge reinforces this interaction by approach of residue Q37 in α2.

In the three structures, two adjacent copies of each complex relate by a 2-fold crystallographic axis, so that two protein molecules sandwich two DNA molecules (Figure 2E). Interestingly, both the hydrophobic wedge and the β-pin interact with the symmetry-related DNA molecule, with each motif contacting DNA from opposite sides of the double helix. The main interaction involves residue H60 in α3 and the DNA region that is most distal from the XPG active site, a contact that is reinforced by K972 in α15 (residues 970–984). Moreover, the α2–α3 loop, which is only partly ordered in one of the six DNA-free molecules, becomes fully ordered in C2b by inserting into the DNA major groove. While density for this loop is weak, residues R43 and R45 lie close to the DNA backbone. Finally, residues N827 and K828 from the β6–β7 loop contact the DNA region that is next to the active site (Figure 2D). As a result, the hydrophobic wedge and the β-pin form a canyon that is rich in basic residues and extends up to helix α15, at the outer rim of the XPG nuclease domain (Figure 2C). In summary, our XPG-DNA structures identify three major DNA-binding areas in XPG: (i) the H2TH motif and α12b for dsDNA; (ii) the active site face of the wedge and the β-pin for the junction; and (iii) the canyon formed by the wedge and β-pin, which could accommodate ssDNA or dsDNA. In addition, while residues K84, K85, R91 and R92 in α4 do not directly contact DNA in our structures, their vicinity to the active site and their location at the inner face of the gateway (Figures 1C and 2C) suggest that they might contact the ssDNA damaged strand (see below).

### Comparison with endonucleases of the flap family

**Figure 3.**
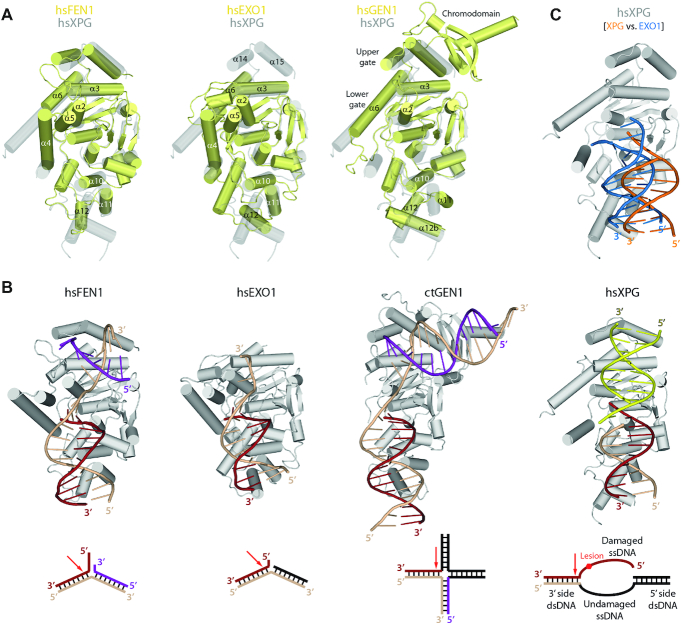
Structural comparison with flap endonucleases. (**A**) Superposition of XPG in transparent grey with FEN1 (PDB: 3Q8M), EXO1 (PDB: 3QE9) and GEN1 (PDB: 5T9J). (**B**) Reported crystal structures of FEN1 (PDB: 3Q8M), EXO1(PDB: 3QE9) and GEN1 (PDB: 5CO8) in complex with DNA, compared with the C1 structure reported here. A schematic of the specific substrate for each protein is shown below. (**C**) Superposition of the XPG-DNA C1 structure in gray and orange onto EXO1-DNA (PDB: 5UZV) in an early pre-incision state, where the EXO1 structure has been removed and its DNA is shown in blue.

We compared the structure of XPG with that of other members in the human structure-specific nuclease superfamily, i.e. FEN1, EXO1 and GEN1. Superposition of XPGn with these proteins showed an overall similarity, while important differences can be identified (Figure 3A, B). The helical arch in XPG presents a unique configuration. While in GEN1 α6 follows a path that is similar to that observed in XPG, GEN1 α6 is shorter and α4 is missing. This leads to the absence of a gateway in GEN1, which rather exhibits upper and lower gates for ssDNA binding (21), likely due to different substrate specificity. Furthermore, when compared with FEN1 and EXO1, the helical arch presents a markedly different configuration, with a significantly longer α6 in XPG and α4 tilted by about 90°. Moreover, helix α5 in FEN1 and EXO1 is replaced by a disordered ∼640-residue spacer in XPG, which determines the specificity of this protein for DNA bubbles instead of shorter 5′ flaps (13). Additionally, the XPG hydrophobic wedge occupies an intermediate position between those observed in GEN1, where it lies further from the helical arch, and FEN1 or EXO1, where it lies closer to the arch. This is likely a consequence of the unique arch configuration in XPG, which allows α2 in the wedge to significantly approach α6 in the arch, suggesting that a crosstalk may operate between these motifs. Besides, while the H2TH motif arranges similar to other human flap endonucleases, the α10–α11 loop lies closer to DNA in XPG, which associates with the absence of a K^+^ ion mediating the interaction. Moreover, the DNA molecule in our C1 structure is tilted by ∼15° away from the active site, as compared to other human flap nucleases. This difference is less pronounced when compared with the structure of an early reaction intermediate of EXO1 (35), suggesting that our structures reflect a stage that precedes DNA cleavage (Figure 3C).

Finally, two additional regions in the protein present remarkable differences with other members of the human endonuclease family. On one side, XPG presents a unique insertion that includes α12b and the α12–α12b loop. While GEN1 contains an insertion in an equivalent position, both the helix and the loop are significantly longer as compared to XPG, and the reported structure lacks DNA in this region (21). On the opposite side, XPG contains helices α14 and α15 at the end of the nuclease domain. Though absent in EXO1, these helices in FEN1 and GEN1 are involved in upstream dsDNA binding. Moreover, GEN1 contains an extra chromodomain that allows binding of longer DNA in this region. In summary, in spite of similarities to other members of the nuclease family, XPG presents unique structural features that are probably crucial in determining its specificity for DNA repair bubbles.

We also compared our structures of XPGn with equivalent structures of its yeast homolog, Rad2. The gateway defined by the XPG helical arch has not been observed in Rad2 (25), as the N-terminal half of α6 appears disordered and tilted away from α4 (Supplementary Figure S5A). This underscores the arch plasticity, presumably related to its putative interaction with the damaged strand ssDNA. Additionally, the wedge helix α2 is tilted away from the active site in activated Rad2 as compared to XPG, associated with a shorter α3 helix. While this could arise from different DNA binding in the compared structures, full ordering of α6 in XPG may determine the conformation of the neighboring wedge helices. Besides, the α10-α11 loop within the H2TH motif is shifted away from DNA by ∼2 Å in Rad2, concomitant with tilting of α11 (Supplementary Figure S5B). Both the loop and α11 establish the main interaction with dsDNA, which in activated Rad2 is mediated by a K^+^ ion that is absent in our XPG structures (see above). A major difference arises around helices α12 and α12b, at the outer rim of the dsDNA-interacting region, which shows poor conservation among orthologues (Supplementary Figure S5C). The DNA-binding α12b is shorter in XPG due to a unique insertion in yeast at the N-terminus of this helix, while the opposite applies to the DNA-distant α12. Between these helices, Rad2 displays an additional helix, α12a, that is absent in our XPG structures. While the XPG α12–α12b loop could fold into a helix as observed for Rad2 (Supplementary Figure S5A), sequence conservation rather supports that α12a is lacking in XPG.

### Model of activated XPG

**Figure 4.**
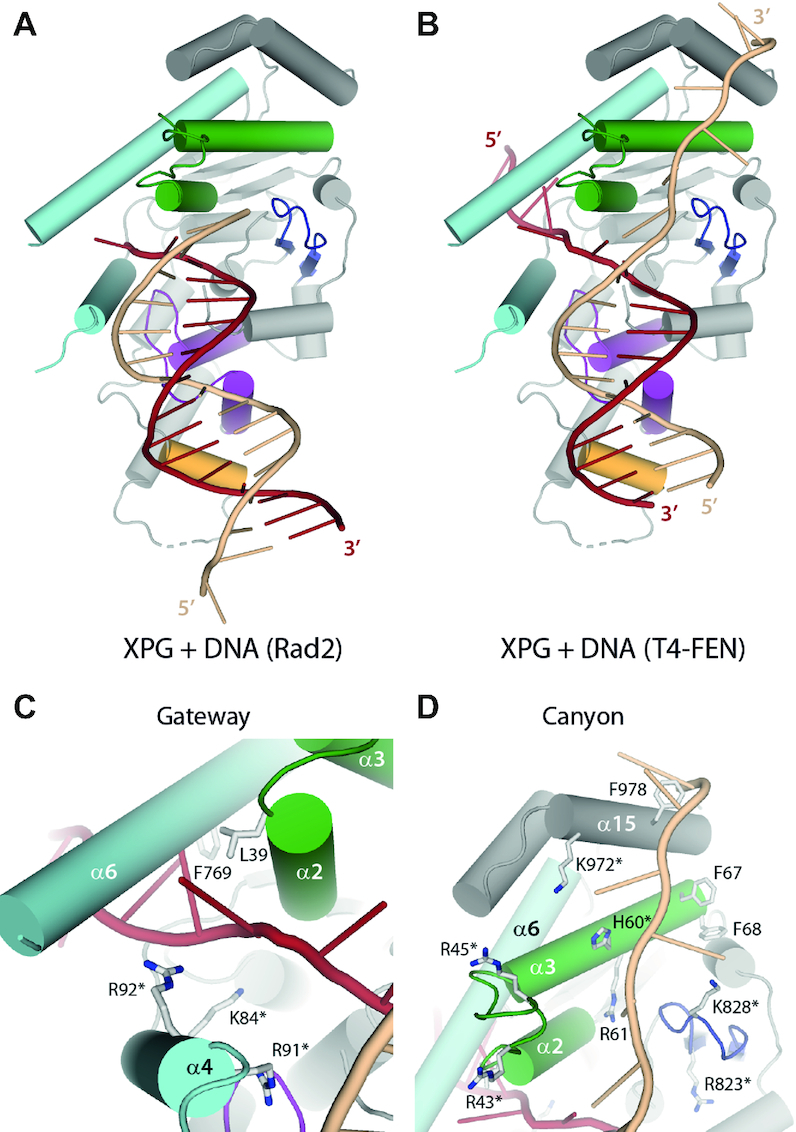
Model of XPG in the activated state. (**A**) Model of activated XPG using the DNA in the Rad2 crystal structure (PDB: 4Q10). (**B**) Model of activated XPG using the DNA in the T4 RNase H crystal structure (PDB 2IHN). (**C**, **D**) Close-up views of the gateway and the canyon from the model shown in panel B. Residues mutated in this work are labeled with an asterisk.

To further infer how XPG may act in the later stages of its catalytic process, we used available structures of flap nucleases in complex with DNA to build a model of activated XPG. We first took the DNA in the Rad2–DNA structure (25) to build a model of XPGn in complex with splayed-arm DNA (Figure 4A). As expected, the damaged strand in the model clashes with the XPG N-terminal methionine, which is absent in the active protein *in vivo*. The undamaged strand exhibits minor clashes with α11 in the H2TH motif, implying small rearrangements in XPG to accommodate DNA before catalysis. Comparison of the model with the structures reported here suggests that DNA accommodation for cleavage requires reorientation of its trajectory, using the H2TH contact as pivoting point (Movie 1). In addition, the model indicates that the unique orientation of α4 in XPG may allow basic residues in this helix, including R91, R94 and K95, to contact the undamaged strand 4–5 nucleotides downstream of the scissile bond. Besides, the model shows that the 5′ flap is positioned exactly at the entrance of the gateway, with residues K84 and R92 in α4 pointing in the direction of the cleaved DNA strand. Interestingly, mutational analysis in the T5 endonuclease showed that K83, equivalent to K84 in XPG (see below), is absolutely required for exonuclease activity while it is dispensable for endonuclease activity (36).

Our structures suggest that the hydrophobic wedge, the β-pin and α15 may play a role in DNA binding beyond the ss/dsDNA junction. To build a putative model of XPGn in complex with the 3′ side of the repair bubble, we used the structure of the FEN1 homolog T4 RNase H, where the path of both ssDNA strands beyond the junction was observed (37). Apart from minor clashes with dsDNA as seen in the Rad2-based model, the ssDNA strands adequately accommodate onto our XPG structure (Figure 4B). The damaged ssDNA engages inside the gateway with only minor clashes with α6, which could be avoided with small rearrangements in the ssDNA and/or the helical arch. This raises the possibility of DNA threading in XPG, as described for FEN1 (38). The presence of basic residues K84 and R92 in α4 and hydrophobic residues L39 and F769 in α6 is compatible with binding to the ssDNA backbone and bases, respectively (Figure 4C). Besides, the undamaged ssDNA fits in the canyon formed by the hydrophobic wedge and the β-pin motifs. The canyon is interspersed with basic residues including H60 and R61 in the wedge helix α3, K828 in the β-pin, and K972 in α15, which could accommodate the phosphate backbone (Figure 4D). In addition, four exposed phenylalanine side chains (F67, F68 and F978) locate in the vicinity of the undamaged strand in our model and could stabilize exposed ssDNA bases. In summary, DNA modelling on our XPGn structures provides hints into DNA binding and cleavage by XPG.

### Structure-based mutational analysis

**Figure 5.**
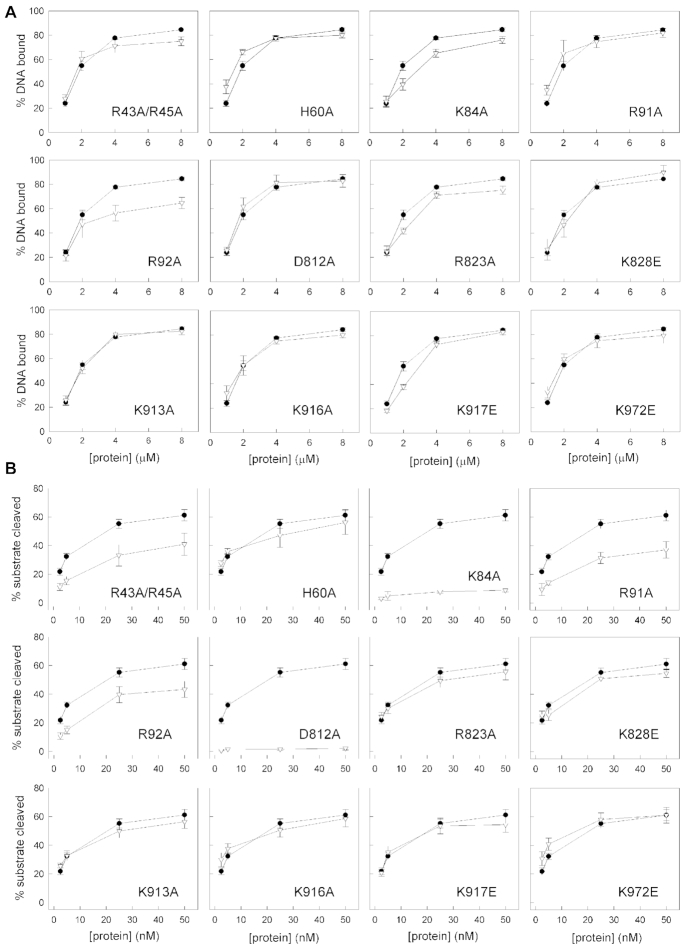
Functional analysis of XPG mutants with splayed-arm DNA. Quantification of DNA-2 (2.5 μM) binding (**A**) and DNA-Y (25 nM) cleavage (**B**) at varying protein concentrations of XPG wild-type (black circles) and point or double mutants (white triangles). Representative gels are shown in Supplementary Figure S6. Graphs represent the quantification of at least three independent experiments, with error bars corresponding to the standard error of each data point.

To identify critical XPG residues for DNA binding and incision, we performed an extensive mutational analysis using EMSA and nuclease activity tests on splayed-arm substrates (Figure 5 and Supplementary Figure S6). We chose to mutate residues that, according to our XPG-DNA structures and model of activated XPG, are likely to play a role in the interaction and/or cleavage of the repair bubble. Mutation of individual basic residues in arch helix α4, which according to our model of activated XPG is expected to interact with the 5′ flap, distinctly affects DNA binding. In particular, R92A decreases DNA binding by about one third, while K84A produces a subtle effect and R91A binds DNA as wild-type XPGn. Nevertheless, mutation of any of these three residues decreases XPG nuclease activity, with R91A and R92A reducing cleavage by half and one third, respectively, while a complete loss occurs in the case of K84A in agreement with mutational analysis of T5 endonuclease (36). The different location of these residues is coherent with their distinct role in DNA binding and cleavage. Notably, K84 lies closest to the active site, while the sidechains of R91 and R92 point towards the entrance and exit of the gateway, respectively (Figures 1C and 4C). Besides, the double mutant R43A/R45A in the hydrophobic wedge loop reduces the nuclease activity to about half, while DNA binding is less affected. The location of this loop between arch helix α6 and the positively-charged canyon is coherent with a role in ssDNA positioning. A second mutant in the hydrophobic wedge, H60A, only exhibits a mild decrease in nuclease activity and no effect in DNA binding. In contrast, mutation of the equivalent position in Rad2 almost entirely suppresses nuclease activity (25), which highlights functional differences between homologues. Additional mutants near the canyon exhibit marginal or no effect in splayed-arm DNA binding or cleavage. R823A in the β-pin shows ∼10% reduction in both assays, while K828E in the β-pin and K972E in helix α15 mildly lower DNA cleavage only (Figures 2D and 4D). On the opposite side of the enzyme, mutants K913A, K916A or K917E in α12b, which binds dsDNA next to the H2TH motif (Figure 2D), also present a minimal or null effect in DNA binding or cleavage. This suggests that, at least individually, these residues have limited influence on DNA positioning, in contrast to Rad2 where the K909A mutant abrogates nuclease activity (25), further underscoring dissimilarities between homologues. Finally, as expected, mutant D812A in the active site has no influence on DNA binding while it completely abolishes nuclease activity. This and other mutants highlight the fact that DNA binding is not sufficient for nuclease activity and indicate that proper DNA positioning is required for cleavage. In conclusion, basic residues in the helical arch and the hydrophobic wedge play key roles in XPG function, while relevant differences are observed between the yeast and human homologs.

### Structural basis of genetic diseases associated to XPG

**Figure 6.**
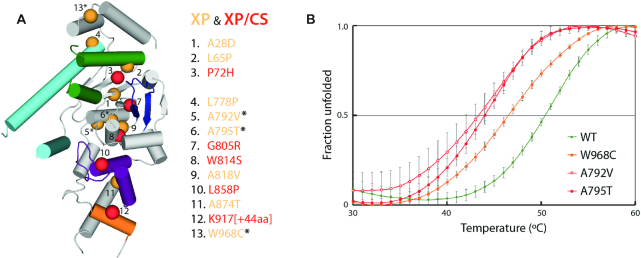
Structural basis of xeroderma pigmentosum (XP) and Cockayne syndrome (CS). (**A**) The location of each mutation is represented as spheres, colored orange for mild phenotypes (XP) or red for severe phenotypes (XP/CS). (**B**) Thermal shift assay for wild-type XPGn (green) and mutant proteins A792V (red open circles), A795T (red closed circles) and W968C (orange), as found in patients. Graphs represent the quantification of three independent experiments, each including a triplicate of every mutant, with error bars corresponding to the standard deviation of each data point. The resulting *T*_m_ values for wild-type, A792V, A795T and W968C are 50.5±0.3, 43.5±1.0, 44.0±0.5 and 46.5±0.5°C, respectively.

The structures of XPGn reported here provide an atomic framework to rationalize XPG mutations found in XP and CS patients (summarized in (39)). From the thirteen different point mutations identified to date, only I290N lies in the spacer region, absent in our constructs (Supplementary Table S1). The remaining twelve localize within XPGn, which allowed mapping on the C2b structure (Figure 6). An additional 44-residue insertion in XPGn leading to disease was also mapped on our structure. Interestingly, all mutations distribute along an axis running in the longest dimension of the protein, suggesting that alterations at peripheral areas affect its DNA repair functionality mildly.

Three mutations cause severe XP/CS phenotypes with early onset, associated with mental retardation since childhood (40,41). They correlate with non-conservative substitutions in the hydrophobic core of the enzyme and are, thus, expected to strongly destabilize the structure, as predicted by DUET (42). P72H likely interferes with the nuclease fold at the base of α3 in the wedge, whereas G805R and W814S affect the structure around the β-pin (Supplementary Figure S7). Interestingly, while G805R likely induces major clashes, W814S is predicted to collapse the hydrophobic core by removal of a large hydrophobic residue. Two other mutations cause severe XP/CS phenotypes with late onset (43). L858P may lead to increased rigidity or disruption of secondary structure at α10 in the H2TH motif, essential for DNA binding and positioning. In contrast, the 44-residue insertion within the unique XPG helix α12b is expected to interfere with dsDNA interaction beyond the H2TH motif.

The remaining point mutations lead to milder phenotypes, with symptoms related to high UV light sensitivity (39,44–48). The conservative L65P mutation may lead to increased rigidity or disruption of secondary structure at α3 in the wedge, which may influence the helical arch configuration (Supplementary Figure S7). An equivalent rigidifying effect at the basis of α6 in the helical arch is expected for L778P. This mutant reinforces the critical role of α6 conformational changes, which we showed to determine the configuration of the gateway. Both A792V and A795T likely shift α7, which may alter the structure around the neighboring active site. A thermal shift assay using purified proteins shows that either of these mutants severely reduces the stability of XPGn by 7 and 6.5°C, respectively (Figure 6B), suggesting that their associated XP phenotype is related to structural instability. While predicted to stabilize the nuclease fold according to DUET, the conservative A818V mutation lies at the basis of the β-pin and may influence its orientation. The A874T mutation is expected to alter the orientation of α11 in the H2TH motif, which plays a prominent role in DNA binding according to the structures reported here.

Finally, two siblings were reported to exhibit a mild XP phenotype with a heterozygous genotype leading to mutated XPG at A28D or W968C (48). According to DUET, both mutations are predicted to destabilize the protein, with A28D exhibiting a stronger effect (Supplementary Table S1). In agreement, our structure shows that A28 lies within the hydrophobic core while W968C locates in the α14-α15 loop at the outer rim of the protein (Figure 6A). Interestingly, the W968 sidechain lies next to DNA and forms a cation-π interaction with K972 (Supplementary Figure S6A), which also locates next to DNA (Figure 2D). Moreover, F316 in FEN1, equivalent to W968 in XPG, is involved in the recognition of the 3′ flap (49). While a 3′ flap is absent in repair bubbles, these observations suggest that the W968C mutation may interfere with DNA binding. To investigate the effect of this interesting mutation in XPG, we expressed and purified the W968C mutant and compared its biochemical properties with those of the wild-type protein. Our thermal shift assay showed that introduction of the W968C mutation leads to a reduction of the melting temperature by 4°C (Figure 6B). These results support that structural instability is at the basis of the XP phenotype due to the W968C mutation.

## DISCUSSION

### The helical arch is a dynamic region forming a gateway

In this work, we present crystal structures of the human XPG nuclease domain, both in the absence and presence of DNA. The nine different atomic models derived from these structures provide hints into the enzyme architecture and dynamism. Several structural elements exhibit flexibility, which is likely relevant for protein function. Movements in the β-pin loop, α11 and α12b are limited to about 3 Å, while the hydrophobic wedge and the α12–α12b loop are essentially disordered, with the exception of C2b where the wedge orders upon insertion into the DNA major groove. Besides, the helical arch exhibits remarkable conformational changes from fully-unfolded α4 and half-unfolded α6 to both completely folded helices. In particular, α6 constitutes a long, mobile α-helix able to form a pore with the uniquely-oriented α4 helix. An equivalent pore constitutes a gateway for 5′ flap threading in FEN1 and EXO1 (24,35), while GEN1 lacks a gateway in accordance with the absence of a 5′ flap in Holliday junctions (21). In the case of XPG, which only cleaves DNA on the 3′ side of the lesion after a 5′ flap containing the damage has been generated by XPF-ERCC1 on the opposite side of the repair bubble (11), two scenarios for damaged ssDNA binding can be envisioned. In the first, the 5′ flap could be threaded through the helical arch pore before the damage strand is cleaved. In the second, the 5′ flap could surround the helical arch, most likely by passing between arch helix α6 and wedge helix α2. Our model of XPG with T4-FEN DNA showing that the 5′ flap can be hosted inside the gateway would favor the first hypothesis (Figure 4C). In this model, both K84 and R92 in arch helix α4 lie close to the 5′ flap ssDNA. Our functional analysis confirms that mutation of these residues lowers binding of splayed-arm DNA to XPG, indicating a role in 5′ flap positioning prior to nuclease attack. Moreover, mutation of K84, R91 or R92 reduces the catalytic activity of the enzyme, with complete loss of function in the case of K84, suggesting that it may play a direct role in catalysis. Additionally, it was shown that 5′ flap nucleotides 1 to 4 from the ss/dsDNA junction are protected in the XPG-DNA complex (34), which is more likely in the first scenario. The high plasticity of the arch helices, connected through a largely-disordered ∼640-residue spacer, is compatible with large conformational changes presumably required for threading. Nevertheless, in contrast to FEN1, the 5′ flap in the XPG substrate is 24–29 nucleotides long (17,18) and threading of this long ssDNA would likely require a massive rearrangement of the repair complex, possibly including TFIIH, which seems unlikely. Moreover, XPG can cleave artificial bubbles lacking a 5′ flap (20), showing that threading is not required for DNA cleavage *in vitro*.

### The H2TH motif allows XPG swiveling on dsDNA

Our crystal structures of XPGn in complex with a short splayed-arm DNA shed light into how this protein binds DNA. The main anchoring point locates at the N-terminus of α11 within the H2TH motif, as observed for other members of the family. Binding of dsDNA beyond this point is reinforced by interaction with α1 and α12b, the latter being unique in XPG and its yeast orthologue, Rad2 (25). Our structures show two strikingly different modes of interaction with dsDNA in this region. The α11 and α12b helices can either bind the minor or the major groove of DNA, with each helix contacting one strand of the groove. The minor groove binding mode likely represents the canonical interaction, as this has been observed in other members of the family (Figure 3). Interestingly, we obtained two different structures of the minor groove binding differing in the tilt angle of dsDNA respect to XPG. This suggests that swiveling of XPG on dsDNA may play a role in protein function. Swiveling is supported by comparison with Rad2-DNA structures in the activated stated, which suggests that pivoting of dsDNA on α11 would position the nucleic acid for nuclease attack (Movie 1). Repositioning of DNA for cleavage likely associates with binding, at the protein–DNA interface, of monovalent cations such as K^+^, like in Rad2 (25), Na^+^, observed in EXO1 (35), or Li^+^, which we report to be as productive as K^+^ for XPG activity (Supplementary Figure S2C). Besides, the major groove binding mode likely represents a non-productive complex. Nevertheless, this peculiar interaction raises the possibility of DNA scanning by sliding on the DNA, rather than by rotation around the DNA minor groove. This is supported by structural studies in EXO1, showing that the H2TH is able to slide by 1 nucleotide, assisted by the monovalent ion mediating the interaction (35).

### The hydrophobic wedge and the β-pin form a DNA-binding canyon

In addition to the main DNA contact point around the H2TH motif, the hydrophobic wedge and the β-pin contact a symmetry-related DNA molecule in our structures. Notably, by inserting into the major groove of dsDNA, the wedge loop orders in a unique configuration. The hydrophobic wedge and the β-pin form a shallow canyon with a positively-charged surface that reaches α15 at the outer rim of XPGn. Modeling of a splayed-arm substrate on our structure suggests that this canyon is the binding site for the undamaged ssDNA strand in the repair bubble (Figure 4D). An open canyon is compatible with experiments showing that the 3′ overhang in splayed-arm substrates, corresponding to the undamaged ssDNA strand in the repair bubble, is not protected by XPG (34). These authors also show that at least two nucleotides of the 3′ overhang are required to retain XPG binding and cleavage. In agreement, our mutational analysis indicates that changes in the vicinity of the ss/dsDNA junction, including R43A/R45A and R823A, mildly decrease DNA binding, while canyon mutants that are more distant to the junction, such as H60A, K828E and K972E, behave like the wild-type protein. Moreover, residues R43 and/or R45 in the wedge loop are relevant for proper DNA positioning before cleavage, while other residues in the canyon have little or no influence on nuclease activity. An equivalent canyon in FEN1 and GEN1 is occupied by dsDNA on the 3′ side of their substrates, which raises the possibility of dsDNA binding on the XPG canyon at a certain stage of the repair process. In principle, XPG could simultaneously bind both dsDNA sides of the repair bubble, in way that resembles that observed in FEN1 or GEN1 (Figure 3). Nevertheless, the presence of TFIIH, XPA and XPF-ERCC1 on the 5′ side of the repair bubble likely occlude XPG access to dsDNA on this side. Alternatively, the canyon may bind dsDNA during initial XPG recruitment to the repair bubble, when XPF-ERCC1 is absent, which may be connected to the stimulatory role of XPG in bubble opening by TFIIH (8).

### Implications for nucleotide excision repair

**Figure 7.**
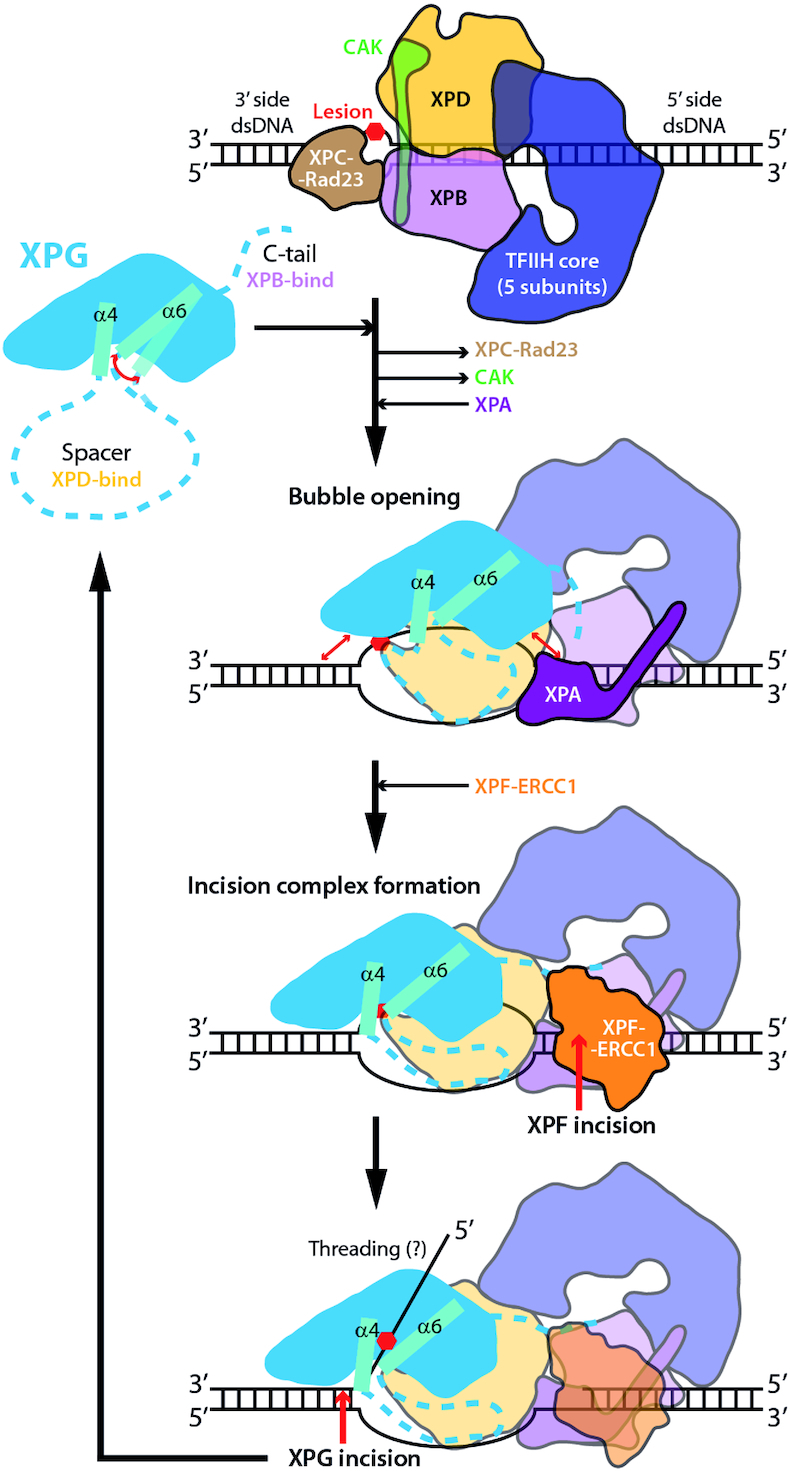
Model of the XPG role in NER. XPG is in blue with arch helices in cyan and regions of unknown structure as dashed lines. Horizontal black arrows denote recruitment or dissociation from the repair complex, while curved red double-arrows indicate mobility and straight red double-arrows indicate putative XPG-DNA interactions. Red single arrows denote incision by XPF-ERCC1 and XPG nucleases.

Based on our results, we propose a model for XPG function in NER (Figure 7). In the first stages, binding of TFIIH on DNA next to the lesion precedes recruitment of XPA and XPG (10). We hypothesize that initial recruitment of XPG implies interaction with dsDNA in a configuration that may mimic that observed in our crystals. In this context, the enzyme likely contacts DNA through its H2TH, assisted by α1 and α12b. This allows XPG to scan the dsDNA on the 3′ side of the lesion, possibly by sliding along one face of the double helix. The canyon formed by the wedge and the β-pin may contact dsDNA on the 5′ side of the lesion, but this remains to be confirmed. At this stage, XPG also binds TFIIH subunits XPB and XPD using its C-terminal tail and the N-terminal third of its spacer region, respectively, as seen by crosslinking experiments (8). The dynamic helical arch likely mediates the transfer of information between the spacer region and the nuclease domain. Therefore, subsequent conformational changes in TFIIH can be sensed by the helical arch, which can transfer the information to the wedge. This likely orchestrates swiveling of XPG on the dsDNA using α11 as pivoting point so that the protein adopts a pre-activated configuration on DNA. Upon DNA cleavage XPF-ERCC1 on the 5′ side of the lesion, further rearrangements of the repair complex likely lead to correct positioning of the undamaged ssDNA strand in the canyon formed by the wedge and the β-pin, and accommodation of the scissile bond near the active site. Whether the long 5′ flap is threaded through the gateway requires further investigation. This activated configuration would lead to XPG-mediated DNA cleavage on the 3′ side of the lesion. Finally, the damaged oligonucleotide is eliminated prior to completion of DNA repair.

### A new framework for therapeutic applications

The crystal structures reported here provide an atomic framework to understand the molecular basis of XP and CS. Severe phenotypes associate with significant structural damage within the XPG hydrophobic core or around the main DNA-interacting region, i.e. the H2TH motif or helix α12b. In contrast, milder phenotypes generally associate with minimal structural damage in those areas, reduced mobility of structural motifs, or reduced protein stability, as we show for mutants A792V, A795T and W968C. Besides, our structures can assist the development of compounds able to modulate the activity of this essential player in the repair of bulky DNA lesions. Notably, XPG levels have been related with the effectiveness of different chemotherapeutic agents used in cancer treatment. Intriguingly, high XPG levels correlate both with sensitivity to trabectedin and with resistance to cisplatin chemotherapy in ovarian, colon and XP cell lines (50). The atomic structures of XPG constitute a valuable tool to design inhibitors that could combined with cisplatin or equivalent drugs in tumors refractory to these compounds.

During final stages of revision of this work, a related study was published (51). This integrated structural study includes the structure of a DNA-free XPG construct, where the XPG helical arch had been replaced by that of *Pyrococcus furiosus* FEN1. This chimeric protein exhibits a fixed orientation for the helical arch and neighboring regions that is distinct from that observed in the DNA-free and DNA-bound XPG structures reported here. Further structure-function studies are now instrumental to deepen our knowledge on the essential role of XPG in DNA repair.

## DATA AVAILABILITY

The atomic coordinates of Free1, Free2, Complex1 and Complex2 were deposited in the Protein Data Bank under accession codes 6TUR, 6TUS, 6TUW and 6TUX, respectively.

## Supplementary Material

gkaa688_Supplemental_Files

## Notes

Present address: Nicholas M. I. Taylor, Novo Nordisk Foundation Center for Protein Research, Faculty of Health and Medical Sciences, University of Copenhagen, Blegdamsvej 3B, Copenhagen 2200, Denmark.
