# Supplementary material for: The crystal structure of human XPG, the xeroderma pigmentosum group G endonuclease, provides insight into nucleotide excision DNA repair
Source: Nucleic Acids Res. 2020 Aug 21;48(17):9943–58. doi: 10.1093/nar/gkaa688 (PMC7515719; doi:10.1093/nar/gkaa688)
Supplement: gkaa688_Supplemental_Files [file gkaa688_supplemental_files.zip › gonzalezcorrochano_supp_movcap.pdf]

The crystal structure of human XPG provides insight into nucleotide excision DNA repair

Rocío González-Corrochano[1], Federico M. Ruiz[1], Nicholas M. I. Taylor[1], Sonia Huecas, Srdja Drakulic, Mercedes Spínola-Amilibia, Carlos Fernández-Tornero[*]

[1]These authors contributed equally to this work

[*]Corresponding author. Email: cftornero@cib.csic.es

****

Supplementary Movie Caption

Movie 1. Morph between the XPG-DNA Complex1 structure and the modelled activated state. The DNA molecule from the C1 complex structure (orange) rotates reaching the geometry observed in the Rad2-DNA complex (blue) and using the H2TH contact as pivoting point. This movement suggests the required reorientation of DNA, after binding to XPG (grey), in order to enter into the active site.
