# Supplementary material for: The crystal structure of human XPG, the xeroderma pigmentosum group G endonuclease, provides insight into nucleotide excision DNA repair
Source: Nucleic Acids Res. 2020 Aug 21;48(17):9943–58. doi: 10.1093/nar/gkaa688 (PMC7515719; doi:10.1093/nar/gkaa688)
Supplement: gkaa688_Supplemental_Files [file gkaa688_supplemental_files.zip › gonzalezcorrochano_sup_rev2.pdf]

Supplementary Data

The crystal structure of human XPG, the xeroderma pigmentosum group G endonuclease, provides insight into nucleotide excision DNA repair

Rocío González-Corrochano[1], Federico M. Ruiz[1], Nicholas M. I. Taylor[1], Sonia Huecas, Srdja Drakulic, Mercedes Spínola-Amilibia, Carlos Fernández-Tornero[*]

[1]These authors contributed equally to this work

[*]Corresponding author. Email: cftornero@cib.csic.es

This file includes:

Supplementary Figures S1 to S7
Supplementary Tables S1 to S2

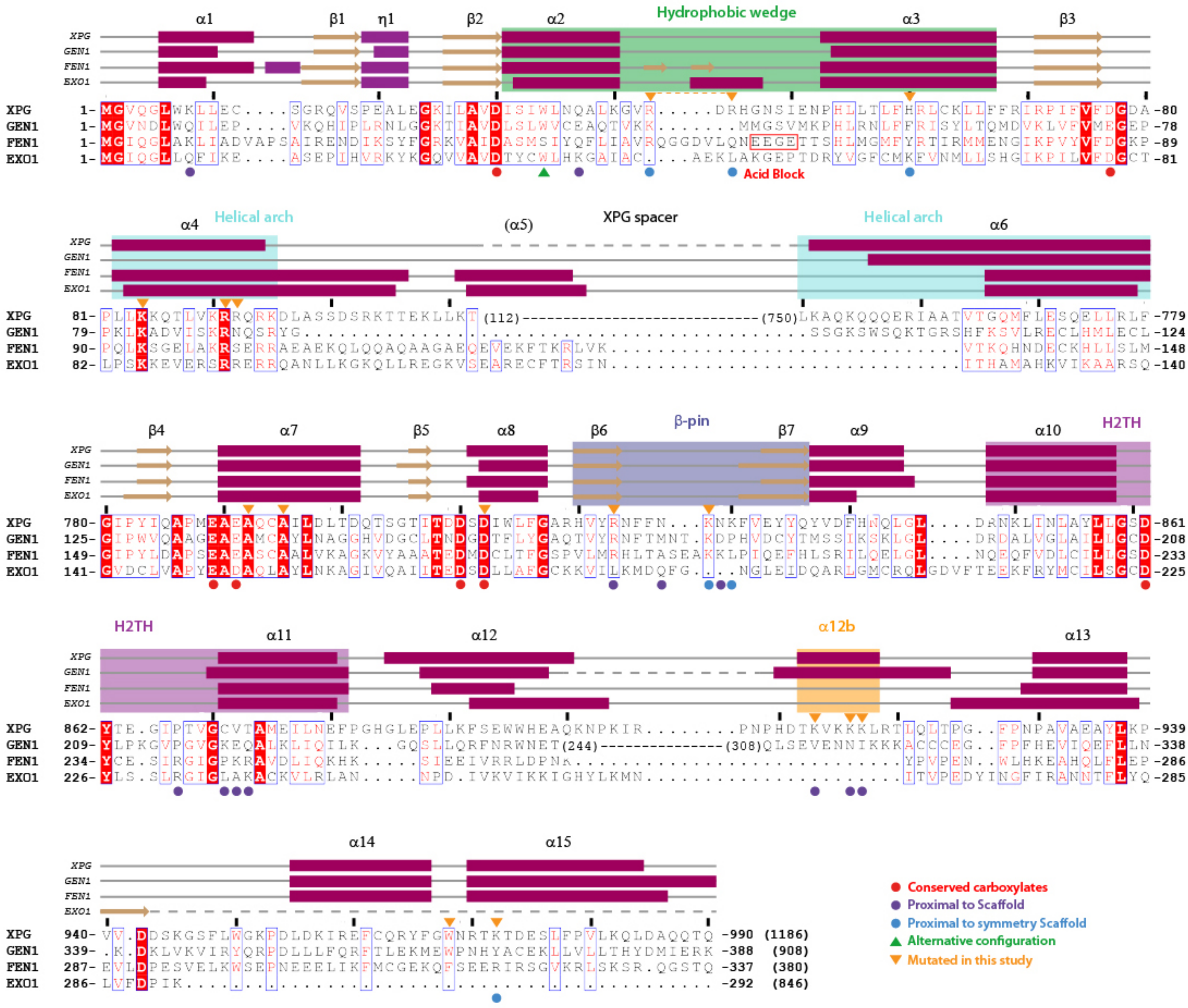

Figure S1. Alignment of nuclease domains from human flap endonucleases. The sequence alignment is based in the structures reported here and elsewhere, and represented with ESPript (http://espript.ibcp.fr). Structural motifs and secondary structure elements are indicated above the alignment, with α-helices in dark red and β-strands in ochre. Disordered or missing parts are represented with dashed lines. Catalytic residues are marked with red circles, while DNA-proximal residues are indicated with purple and blue circles. Mutated residues in this work are marked with an orange triangle above the sequence, with the double mutant R43A/R45A indicated with two triangles connected by a dashed line.

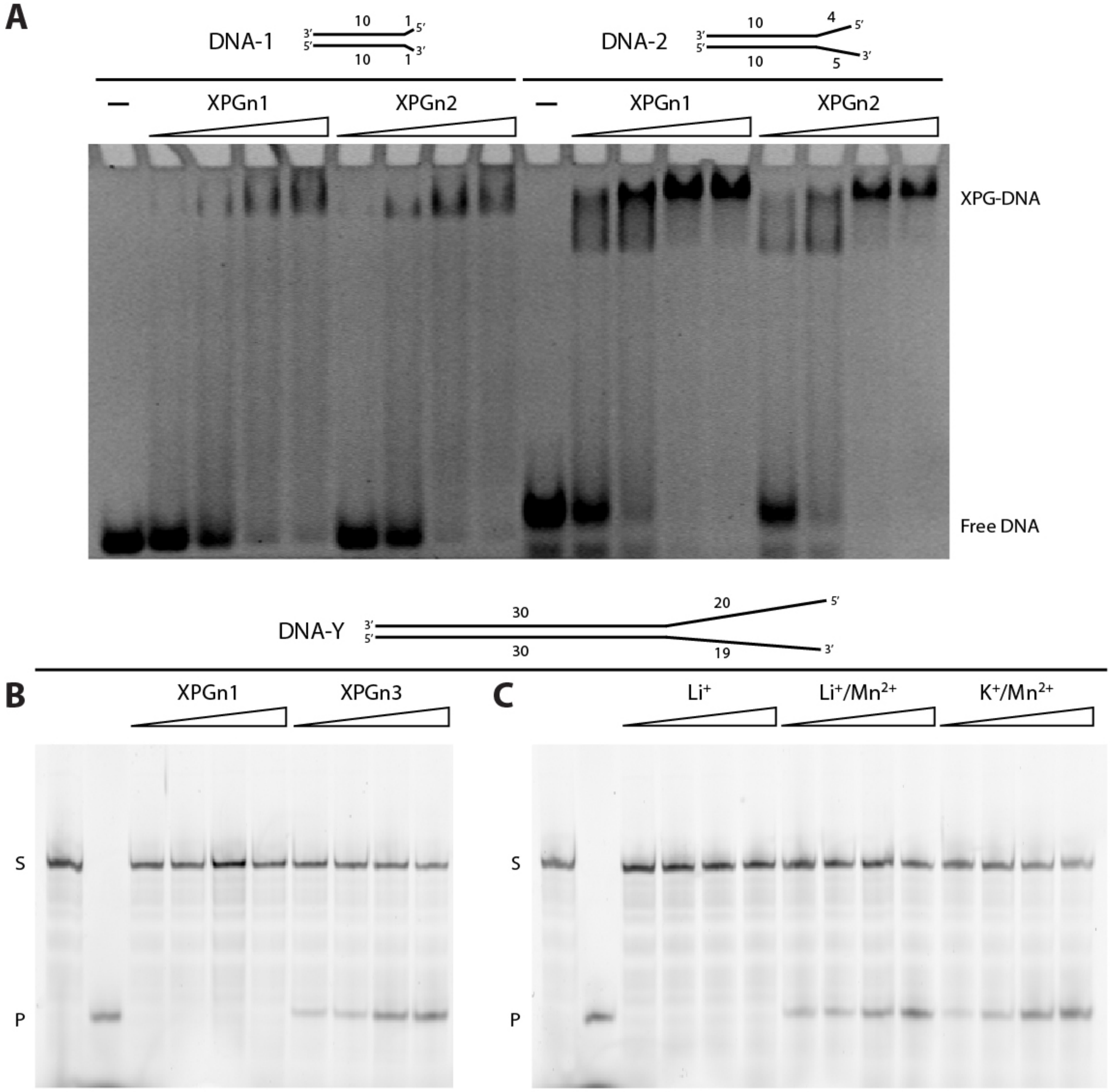

Figure S2. Functional analysis of XPG constructs. (A) EMSA for crystallized constructs XPGn1 and XPGn2 with crystallized DNA-1 and non-crystallized DNA-2. 2.5 μM of DNAs were incubated with increasing protein concentrations (1, 2, 4 and 8 μM). Control lanes 1 and 10 lacking the protein are marked with a dash. (B) Nuclease activity test for wild-type XPGn1 and XPGn3, which include and lack the N-terminal Met, respectively. 25 nM of Cy5-labelled DNA-Y was mixed with increasing concentrations of the enzyme (2.5, 5, 25 and 50 nM). Lanes 1 and 2, controls of the substrate (S) and resulting product (P), respectively. (C) Nuclease activity test for wild-type XPGn3 in the presence of different cations. The experiment conditions and control lanes are the same as in panel B.

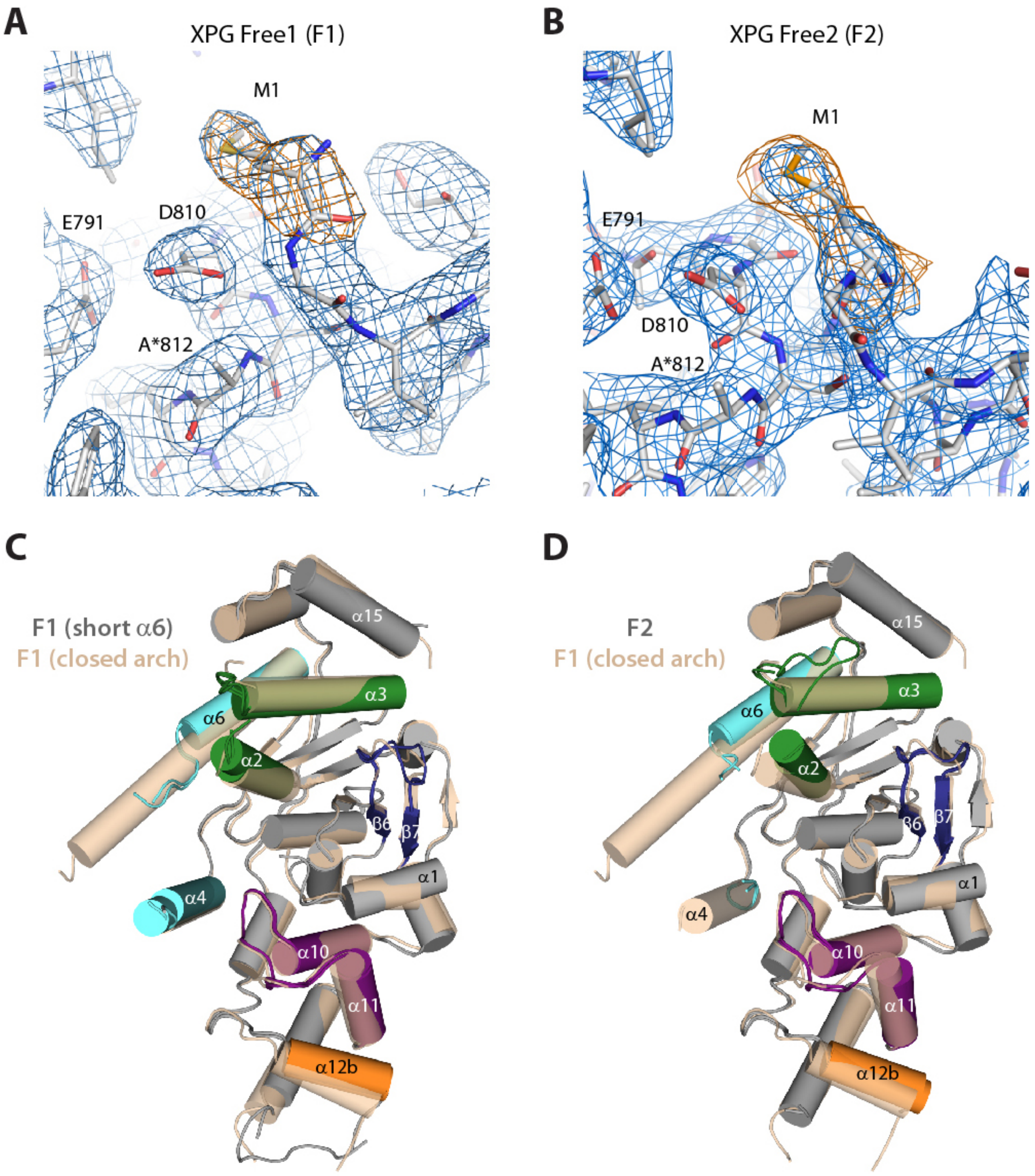

Figure S3. Crystal structure of XPGn in the DNA-free state. (A and B) 2Fo-Fc map of F1 (A) and F2 structures contoured at 1σ in blue, with omit map contoured at 3σ around residue Met1 in orange. The map confirms the D812A mutation. (C and D) Superposition of the F1 molecule with closed helical arch, colored cream, and the two F1 molecules with short α6 (C) or the two F2 molecules (D).

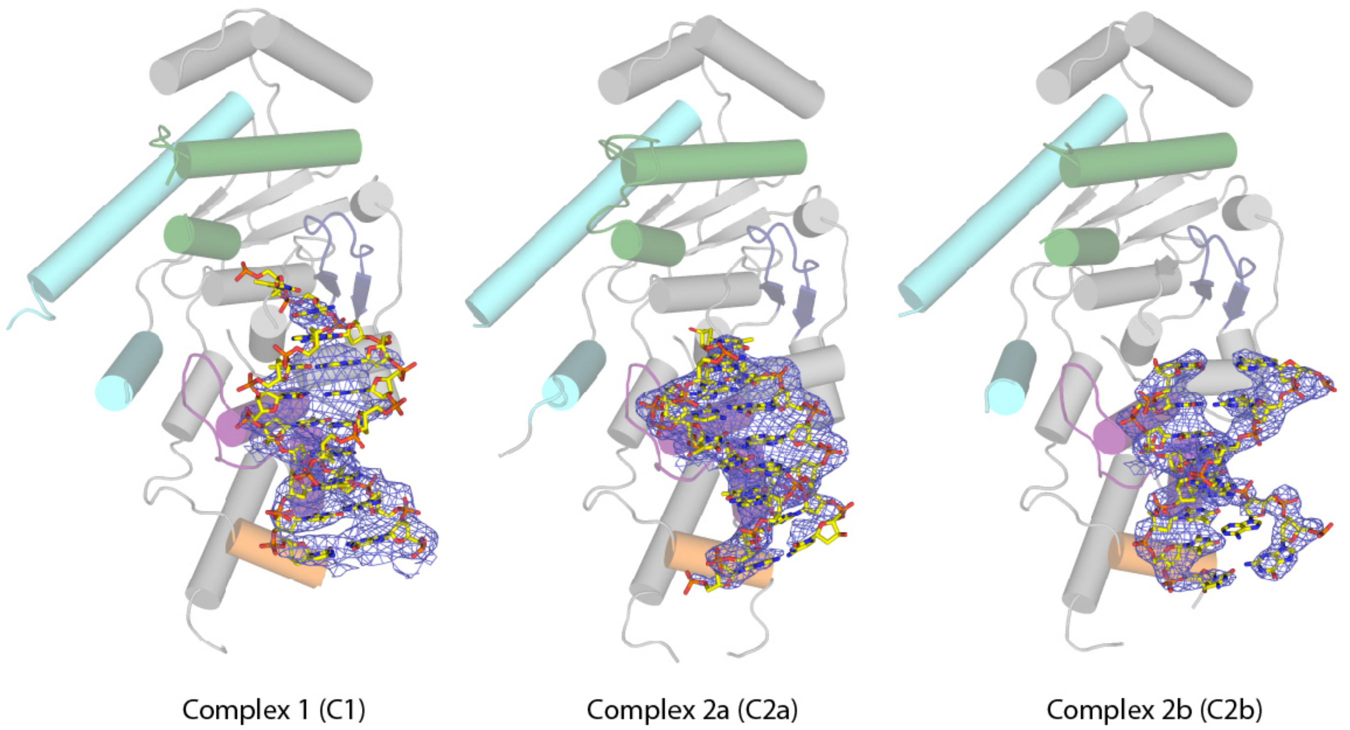

Figure S4. Crystal structure of XPGn in complex with DNA. Structures of XPGn in complex with DNA showing the 2Fo-Fc map around DNA in blue.

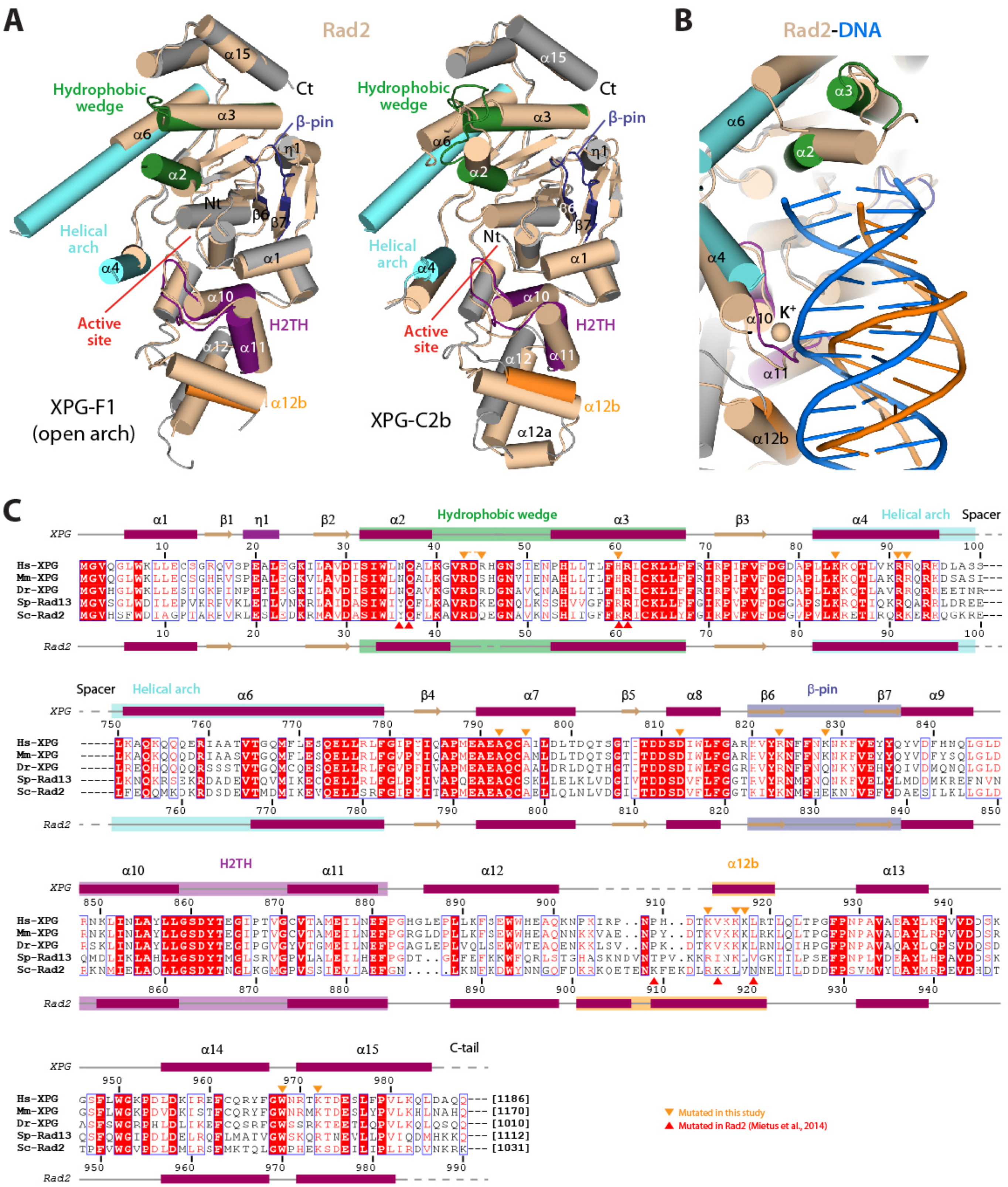

Figure S5. Structural comparison between XPG and Rad2. (A) Superpositions of F1 with open helical arch (left) and C2b (right) with the corresponding Rad2 structures (PDBs 4Q0R and 4Q0W, respectively) in cream color. (B) Close-up view of the DNA-binding region in the superposition shown on the right side of panel A. Rad2 and XPG DNAs are in blue and orange, respectively. (C) Alignment of nuclease domains from different XPG homologs, represented with ESPript (http://espript.ibcp.fr). Structural motifs and secondary structure elements in XPG and Rad2 (25) are indicated above and below the alignment, respectively. Disordered or missing parts are represented with dashed lines. Mutated residues in this study and for Rad2 (25) are marked with an orange and red triangles above and below the sequence, respectively.

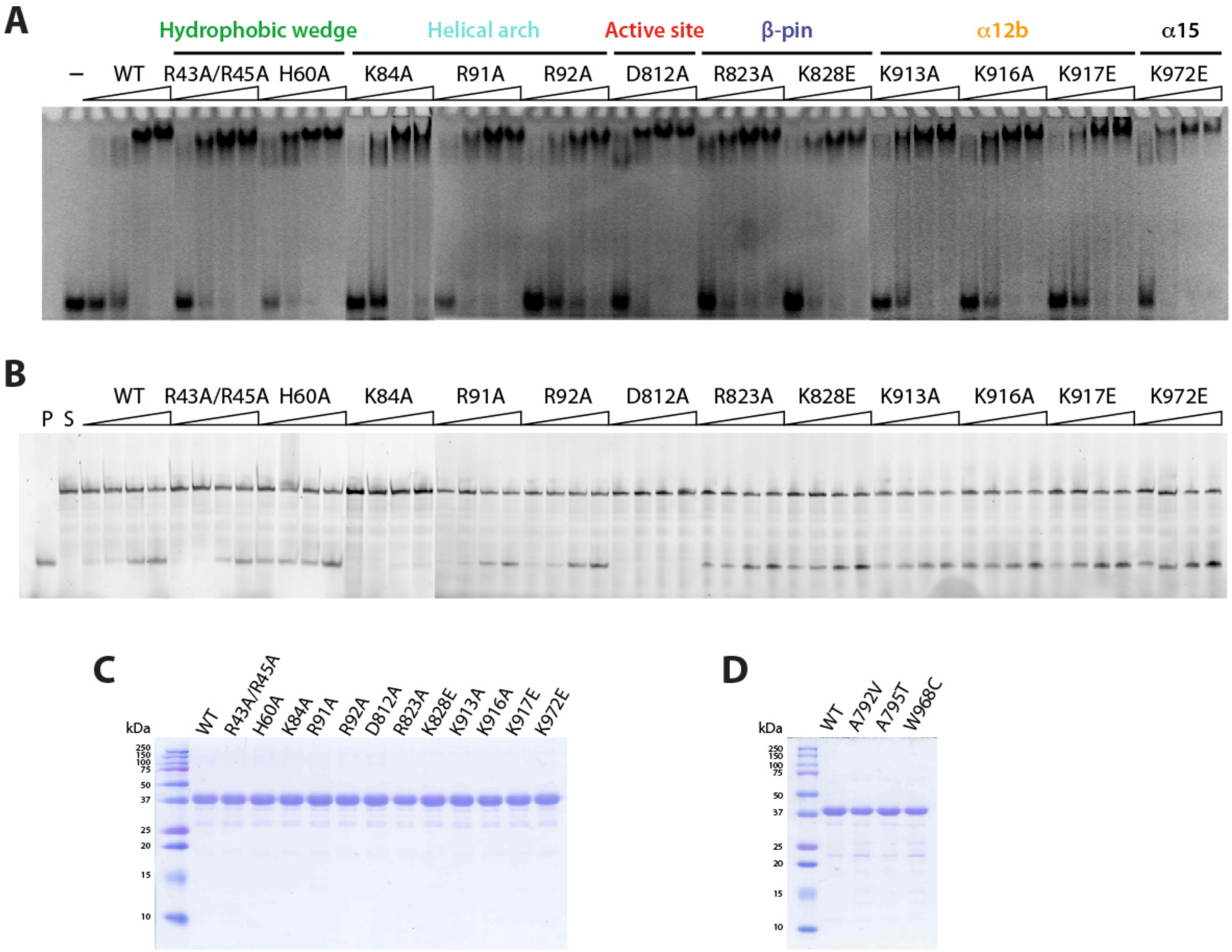

Figure S6. Functional analysis of XPG mutants with splayed-arm DNA. (A) DNA binding to XPG wild-type and point mutants analyzed using EMSA. 2.5 µM of substrate DNA-2 was incubated with increasing protein concentrations (1, 2, 4 and 8 µM). Control lane 1 lacking the protein is marked with a dash. (B) Nuclease activity assay for XPG wild-type and mutants. 25 nM of Cy5-labelled of DNA-Y was mixed with increasing concentrations of the enzyme (2.5, 5, 25 and 50 nM). Lane 1 and lane 2 are controls of the reaction product (P) and substrate (S). For both panels, quantifications of at least three experiments are shown in Figure 5. (C, D) SDS-PAGE analysis of purified proteins (1 µg) used in EMSA and nuclease activity tests (C) or in the thermal shift assay (D). Lane 1 contains molecular weight standards.

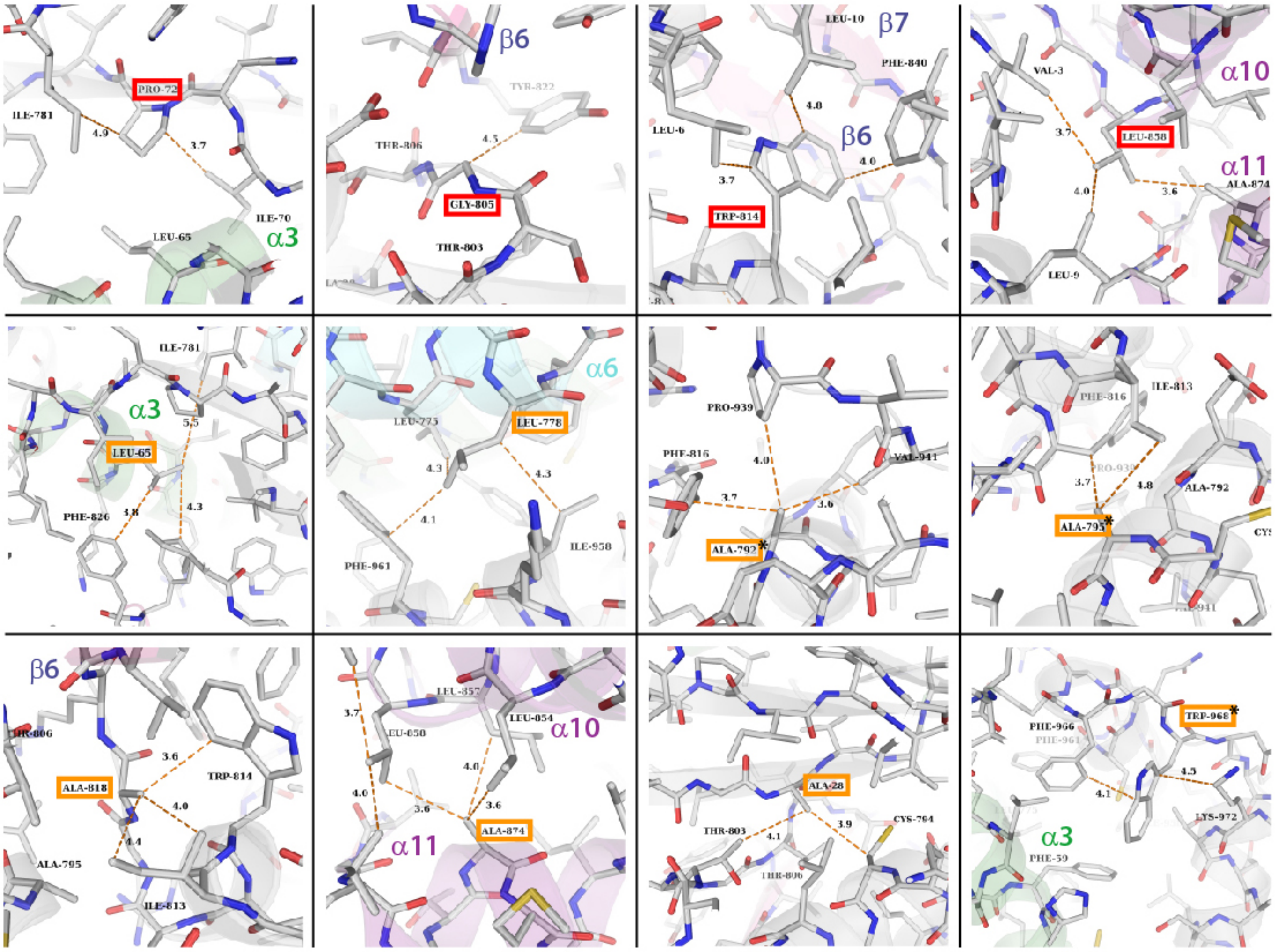

Figure S7. Structural basis of xeroderma pigmentosum (XP) and Cockayne syndrome (CS). Atomic environment of the mutated residues found in patients. Red and orange lines boxing the mutated residue labels are depicted for severe and mild phenotypes, respectively. Residues A792, A795 and W968, mutated in this study, are marked with an asterisk.

Table S1. Summary of described XPG mutations not leading to truncated versions of the protein.

	Mutation	Alleles*	Phenotype	Location	DDG (Kcal/mol)	Effect	Reference
**1**	A28D	Heterozygous (W968C)	XP	β2	-2.42	Destabilizing	Soltys et al., 2013
**2**	L65P	Homozygous	XP	α3	-1.63	Destabilizing	Moriwaki et al., 2012
**3**	P72H	Heterozygous	XP/CS (early)	β3	-1.95	Destabilizing	Zafeiriou et al., 2001
**4**	I290N	Homozygous	XP	Spacer region**	N/A	N/A	Fassihi et al., 2016
**5**	L778P	Homozygous	XP	α6	-1.31	Destabilizing	Chikhaoui et al., 2019
**6**	A792V	Heterozygous	XP	α7	-0.33	Destabilizing	Nouspikel & Clarkson, 1994
**7**	A795T	Heterozygous	XP	α7	-2.08	Destabilizing	Fassihi et al., 2016
**8**	G805R	Homozygous	XP/CS (early)	β5	-1.34	Destabilizing	Schäfer et al., 2013
**9**	W814S	Heterozygous	XP/CS (early)	α8	-2.21	Destabilizing	Schäfer et al., 2013
**10**	A818V	Heterozygous	XP	α8-β6 loop	0.07	Stabilizing	Fassihi et al., 2016
**11**	L858P	Heterozygous	XP/CS (late)	α10	-2.183	Destabilizing	Lalle et al., 2002
**12**	A874T	Heterozygous	XP	α11	-2.093	Destabilizing	Emmert et al., 2002
**13**	K917[+44aa]	Heterozygous	XP/CS (late)	α12	N/A	N/A	Lalle et al., 2002
**14**	W968C	Heterozygous (A28D)	XP	α14-α15 loop	-1.379	Destabilizing	Soltys et al., 2013

*Unless indicated in parenthesis, heterozygous alleles appear in combination with alleles encoding for truncated versions of the protein

**Absent in the crystal structures reported here

Table S2. Oligonucleotides.

Name	Sequence
DNA-1a	5’-TTGCAGAGTTC-3’
DNA-1b	5’-GAACTCTGCAG-3’
DNA-2a	5’-TACTTGCAGAGTTCGC-3’
DNA-2b	5’-GCGAACTCTGCAGAGCA-3’
DNA-Ya	5′-(Cy5)TCAAAGTCACGACCTAGACACTGCGAGCTCGAATTCACTGGAGTGACCTC-3’
DNA-Yb	5′-GAGGTCACTCCAGTGAATTCGAGCTCGCAGCAATGAGCACATACCTAGT-3’
DNA-Yp	5′-(Cy5)TCAAAGTCACGACCTAGACA-3’
